# Competitive cleaning: behavioural variation supports coexistence of two juvenile sympatric cleanerfishes

**DOI:** 10.1098/rsbl.2025.0079

**Published:** 2025-07-16

**Authors:** Rohan M. Brooker, Zara-Louise Cowan, Jake Martin, Sylvie Evans, Bob B. M. Wong, David Lecchini, William E. Feeney

**Affiliations:** ^1^ Australian Institute of Marine Science, Indian Ocean Marine Research Centre, The University of Western Australia, Crawley, Western Australia, Australia; ^2^ School of Life and Environmental Sciences, Deakin University, Geelong, Victoria, Australia; ^3^ Department of Biological and Environmental Sciences, Kristineberg Center for Marine Research and Innovation, University of Gothenburg, Fiskebäckskil, Sweden; ^4^ Department of Wildlife, Fish, and Environmental Studies, Swedish University of Agricultural Sciences, Umeå, Sweden; ^5^ School of Biological Sciences, Monash University, Melbourne, Victoria, Australia; ^6^ PSL Research University, EPHE-UPVD-CNRS, USR3278 CRIOBE, Moorea, French Polynesia; ^7^ Laboratoire d'Excellence "CORAIL", Moorea, French Polynesia; ^8^ Doñana Biological Station, Spanish National Research Council (CSIC), Seville, Spain

**Keywords:** coral reefs, *Labroides*, mutualisms

## Abstract

Cleanerfish support coral reef ecosystems by providing important services to client fishes. Cleanerfish species often coexist, but how interspecies dynamics influence cleaning networks across life-history stages remains largely unknown. Here, we investigated the effects of interspecific competition on the behaviour of two juvenile cleaner wrasses, *Labroides dimidiatus* and *Labroides bicolor*. Under competitive scenarios, *L. dimidiatus* exhibited higher levels of client association compared to *L. bicolor*, while no differences were observed under non-competitive conditions. These behavioural differences likely facilitate resource partitioning and coexistence, supporting the stability of coexisting mutualistic networks in coral reef ecosystems.

## 1. Introduction

Mutualistic interactions can play a fundamental role in shaping communities and maintaining biodiversity within ecosystems [1,2]. On coral reefs, cleaning interactions—where one organism (the cleaner) removes ectoparasites, mucus or dead skin from another (the client)—represent a diverse and widespread mutualism that benefits clients through improved health and fitness, while providing cleaners with a reliable source of food and protection [3]. Cleaning interactions, in turn, have flow-on positive effects on the broader reef environment, promoting biodiversity, shaping habitat structure and enhancing overall resilience [4,5]. While a diverse range of reef fishes and shrimps obligately or facultatively clean [6], cleaner wrasses (*Labroides* spp.) exhibit diverse life-history strategies, spanning the maintenance of highly visible cleaning stations that attract a diverse array of client species [4,7], to roaming throughout larger territories [8]. The frequent distributional overlap of cleanerfishes, with species often occurring within the same community [9], suggests that factors such as competition and niche partitioning may mediate cleaning behaviour, client interactions and the ecological outcomes of these mutualisms.

Coral reef environments are inherently competitive, with high biodiversity and limited resources driving interactions within and among species [10]. For sympatric cleanerfish, the co-occurrence of multiple individuals or species within an area introduces the potential for both intra- and interspecific competition, which can influence behaviour, the frequency and diversity of client associations and the overall structure and function of cleaning mutualisms [9,11,12].

For sympatric cleanerfish, species-specific behavioural traits likely help reduce competition for clients. For example, adult bluestreak cleanerfish (*Labroides dimidiatus*; hereafter referred to as ‘bluestreak’) typically maintain cleaning stations within well-defined home ranges [12,13], whereas bicolor cleanerfish (*Labroides bicolor;* hereafter referred to as ‘bicolor’) are generally less site-attached, attending to clients across larger areas [8,12]. However, direct interspecific competition for clients may be more pronounced during early life stages, as juveniles typically establish small individual cleaning stations close to the reef following settlement, often in the vicinity of other individuals [11]. At this stage, general similarities in habitat use between species heighten the potential for competition, suggesting that subtle behavioural differences may enable individuals to coexist and partition resources effectively. To investigate how behavioural strategies facilitate coexistence in shared habitats, we compared the responses of juvenile bluestreak and bicolor cleanerfish to a model client in the presence of either an interspecific competitor or a non-competitor, assessing the time spent near the client and the frequency of client interactions.

## 2. Methods

The experiment was conducted in November 2019 at the Centre de Recherches Insulaires et Observatoire de l’Environnement (CRIOBE) in Moorea, French Polynesia (17°31′06.9″ S, 149°50′59.4″ W). Three species of juvenile wrasse (Labridae) were used: bluestreak and bicolor cleanerfishes and the clown coris (*Coris aygula;* hereafter referred to as ‘coris’). All three species commonly co-occur on shallow fringing reefs surrounding Moorea, with juveniles of both cleaners actively maintaining small cleaning stations, whereas the coris is a non-cleaning generalist invertivore [14]. Individuals (total length: 3−4 cm; *n* = 20 per species) were collected on either SCUBA or snorkel, using hand nets and a 1 : 3 : 7 clove oil : ethanol : seawater mixture, from two sites: Cook’s Bay reef pass (17°29′16.88″ S, 149°49′7.67″ W) and Temae Reef (17°29′49.56″ S, 149°45′13″ W). In addition, the striated surgeonfish (*Ctenochaetus striatus*; Acanthuridae; hereafter referred to as ‘surgeonfish’) was used as a model client in trials. Individual surgeonfish were collected on snorkel using a barrier net from 'Ōpūnohu Bay (17°29′20.2″ S, 149°51′21.5″ W). This species was selected as surgeonfishes represent clients for both species of cleaner, with each spending a similar proportion of time (approx. 40%) cleaning them [11].

**Figure 1.**
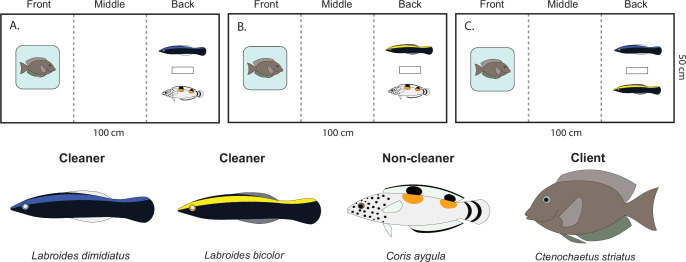
Experimental set-up showing trials where (A) a bluestreak cleanerfish, *Labroides dimidiatus* interacted with the client, the surgeonfish *Ctenochaetus striatus*, in the presence of a non-competitor, the clown coris *Coris aygula* (*n* = 10), or (B) a bicolor cleanerfish, *L. bicolor*, interacted with the client in the presence of the non-competitor (*n* = 10), and (C) a bluestreak and bicolor cleaner interacted with the client in each other’s presence. Stippled lines delineate zones (front, middle and back) in relation to proximity to the client fish. Rectangle in back zone indicates the position of pipe section. Client fish were held in a clear bag, indicated by the blue square, to prevent direct interactions. Fishes are not shown to scale.

Following collection, all fishes were transported to CRIOBE and held for a minimum of 24 h prior to testing. Juveniles were housed in mixed-species groups across several large flow-through holding tanks 2 × 1 × 1 m). Each tank provided ample shelter, including lengths of PVC pipe and pieces of coral rubble, allowing individuals to avoid aggressive interactions and minimize stress. Juveniles were fed *Artemia* to satiation twice daily. Surgeonfish were housed separately in species-specific flow-through tanks of the same dimensions and provided with freshly collected coral rubble covered in turf algae and detritus for food. All fish were monitored closely, and strong feeding responses were taken as an indication of successful acclimation to aquarium conditions. Water quality in all tanks was maintained via continuous flow-through seawater and supplementary aeration. Each individual was used in only one trial, and all fish were returned to their reef of origin following testing. We conducted a behavioural experiment to test if responses to a client varied between cleaner species in the presence or absence of an interspecific competitor. This experiment consisted of three treatments where combinations of two interspecific individuals: (i) bluestreak (cleaner) and coris (non-cleaner); (ii) bicolor (cleaner) and coris (non-cleaner); and (iii) bluestreak (cleaner) and bicolor (cleaner), were exposed to a client (surgeonfish) and subsequent behaviour was assessed (*n* = 10 per treatment; figure 1).

Trials were conducted in white flow-through Plexiglass tanks (length × width × height: 1 × 0.5 × 0.4 m; figure 1). At one end of the tank, a 50 mm section of 15 mm diameter PVC pipe was placed centrally to provide a refuge. In each trial, the two juvenile fishes were introduced into the tank and left to acclimate for 50 min with the water flow on. At 50 min, the water was turned off, and fish were left to acclimate for a further 10 min in still water. At 60 min, the client was carefully introduced to the end of the tank opposite the pipe section. Each client fish was held in a clear polyethylene bag (3.5 l capacity) filled with fresh, aerated seawater. These bags had a flat base and maintained a rigid, upright columnar shape in the water column, allowing the client to move freely within the bag while ensuring it remained in a fixed location during each trial. The client fish maintained regular ventilation rates and natural orientation throughout, indicating minimal stress. Following the introduction of the client, the trial ran for a subsequent period of 5 min with the entire process recorded from directly overhead using a video camera (GoPro Hero9 Black). Camera function was conducted remotely to reduce any disturbance to fishes due to the presence of the operator.

Following completion of all trials, video scoring was conducted using the Behavioural Observation Research Interactive Software (BORIS [15]). For each 5 min recording, two behavioural measurements were scored for each of the juvenile individuals present in the trial: time spent near the client and number of interactions with the client. To assess time near the client, the tank was divided along its length into three zones (front, middle and back zones), each approximately 33 cm. The front zone contained the bagged client, with the middle and back zones increasing in distance from it. For each individual, the proportion of the trial spent in each zone was recorded. A client association score was then calculated by summing the weighted proportion of time the focal fish spent within each of the three zones (i.e. [proportion of time in the front zone × 1] + [proportion of time in the middle zone × 0] + [proportion of time in the back zone × −1]). This score indicates the use of the entire tank relative to the position of the client fish, with a higher score indicating an individual that spent more time near the client (maximum: 1, minimum: −1).

The number of interactions with the client was classified as the number of distinct incidences where an individual moved to within one body length of the bag and was facing the client. Competitive interactions, classed as all behaviours directed towards the other juvenile, e.g. chases and bites, were also scored during data extraction; however, there was an insufficient number recorded to allow inclusion in the subsequent analysis.

To compare the number of interactions with the client fish and the weighted association score a generalized linear mixed effect (GLME) model and a linear mixed effect (LME) model were used, respectively. Models included the competitive scenario—bicolor–coris, bicolor–bluestreak, bluestreak–coris, bluestreak–bicolor, coris–bicolor and coris–bluestreak, with the first species name corresponding to the measured values (i.e. focal fish) and the second species name corresponding to its competitor—as a single fixed effect, and the pair ID (i.e. 1−30) as a random intercept. The resulting models included several pairwise contrasts that were not of interest to the experimental questions, and therefore, only four planned pairwise comparisons were investigated and reported (listed in table 1). These planned comparisons included those within each of the two cleaner species under competitive and non-competitive scenarios (i.e. bicolor with coris to bicolor with bluestreak and bluestreak with coris to bluestreak with bicolor), and those across cleaner species under competitive and non-competitive scenarios (bicolor with coris to bluestreak with coris and bicolor with bluestreak to bluestreak with bicolor). All estimated means and comparisons can be found in the electronic supplementary material [16]. To account for zero-inflation in the count data, we used a zero-inflated Poisson generalized linear mixed effect model.

**Table 1.** Overview of planned contrasts.

within cleaner species contrasts		
bicolor competitive trial (bicolor with bluestreak)	versus	bicolor non-competitive trial (bicolor with coris)
bluestreak competitive trial (bluestreak with bicolor)	versus	bluestreak non-competitive trial (bluestreak with coris)

across cleaner species contrasts		
bicolor competitive trial (bicolor with bluestreak)	versus	bluestreak competitive trial (bluestreak with bicolor)
bicolor non-competitive trial (bicolor with coris)	versus	bluestreak non-competitive trial (bluestreak with coris)

## 3. Results

**Figure 2.**
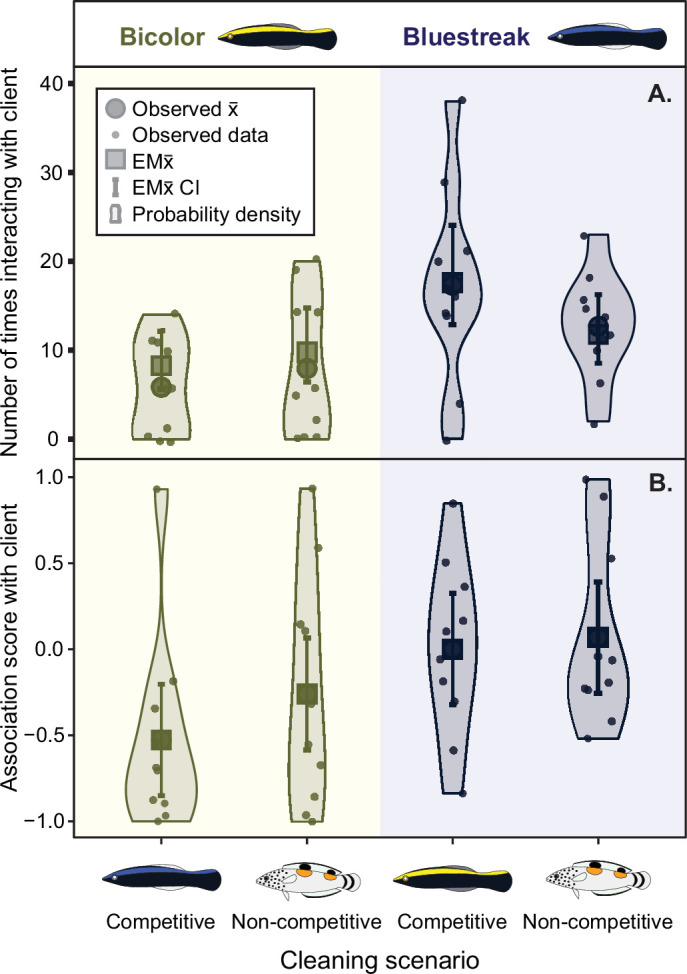
(A) Number of times spent interacting with the client fish under competitive (i.e. paired with a cleaner heterospecific) or non-competitive (i.e. paired with a non-cleaner heterospecific) scenarios across bicolor and bluestreak cleanerfishes. (B) Association score with client under competitive (i.e. paired with a heterospecific cleaner) or non-competitive (i.e. paired with a non-cleaner heterospecific) scenarios across bicolor and bluestreak cleanerfishes.

Under competitive conditions (i.e. a bluestreak and biocolor paring), bluestreaks interacted with the client fish almost 50% more often than bicolors (mean estimate ratio = 0.47, asymptotic CI = 0.316−0.711, adjusted *p* < 0.001; figure 2 and table 2), and spent more time near the client, as indicated by a higher client association score (estimate = 0.53, CI = 0.03−1.03, adjusted *p* = 0.033; figure 2 and table 2). In contrast, there were no statistically significant differences in the number of interactions with the client or client association scores under non-competitive scenarios for either species (both *p* > 0.05; figure 2 and table 2).

**Table 2.** Results of planned comparisons from (A) LME model comparing the client association score across the different focal-fish parings, and (B) GLME comparing the total number of cleaning events across the different focal-fish parings. Adjusted *p*-values have been calculated using a Dunn–Šidák adjustment for four tests (i.e. planned comparisons).

		(A) LME				(B) GLME		
contrast		estimate	*t*-value	*p*-value	adj. *p*-value	estimate means ratio	estimate asymptotic CI	*p*-value
bicolor competitive	bicolor non-competitive	−0.27	−1.181	0.243	0.672	0.852	0.419−1.731	0.967
bluestreak competitive	bluestreak non-competitive	−0.06	−0.299	0.766	0.997	1.491	0.845−2.632	0.283
bicolor non-competitive	bluestreak non-competitive	−0.32	−1.446	0.154	0.489	0.829	0.431−1.598	0.926
bicolor competitive	bluestreak competitive	−0.53	−2.851	0.008	0.033	0.474	0.316−0.711	0.000

## 4. Discussion

Our data suggest that observed differences in client association under interspecific competitive conditions may reflect species-specific behavioural strategies that begin during the juvenile life stage and persist into adulthood. Juvenile bluestreaks tend to clean a broader range of clients, while bicolor juveniles specialize more heavily on certain groups, such as holocentrids found in internal reef cavities [11]. These client preferences may reduce client overlap between the two species and minimize competition at this stage. However, when direct competition is unavoidable, bluestreaks appeared more assertive, spending more time near clients and tending to interact with them more frequently, which aligns with its known dominance and territorial behaviour as an adult [12,17]. In contrast, the lower client association exhibited by bicolors in competitive scenarios may indicate an avoidance strategy, consistent with its adult tendency to rove across broader areas to service clients. Future studies that compare both intra- and interspecific competition may help clarify the degree to which these behavioural patterns are specific to between-species encounters.

These life stage-specific differences suggest that the competitive interactions observed during juvenile stages may shape the cleaning niches of adults, with adult bluestreaks undertaking a ‘territorialist’ strategy, cleaning a wide variety of clients, including many smaller individuals, from fixed stations, while adult bicolors undertake a ‘roving’ strategy, servicing a less diverse client base often comprised of larger individuals [11,12]. Such behavioural plasticity is critical for reducing resource overlap and promoting coexistence within shared habitats. Furthermore, the ability of cleanerfish to adjust their activity in response to competition underscores the role of behaviour in maintaining cleaning networks on coral reefs. As the resilience of these networks supports community dynamics and reef health [4,5], early behavioural divergence may determine how central or peripheral a species becomes within these networks, and its subsequent contribution to ongoing network cohesion [18].

## Data Availability

All supporting data and code for this manuscript are publicly available from the Dryad Digital Repository [16].
